# A simple and reliable microfabrication process for a programmable microvalve array

**DOI:** 10.1016/j.mex.2022.101860

**Published:** 2022-09-16

**Authors:** Zachary Estlack, Beau Compton, Md Enayet Razu, Jungkyu Kim

**Affiliations:** a The University of Utah, United States; b Texas Tech University, United States

**Keywords:** Microvalve design, Microvalve array fabrication, PDMS soft-lithography, Microcontact printing, Capillary electrophoresis integration

## Abstract

We describe our reliable methodology for fabricating a complex programmable microvalve array (PMA) and its integration with a glass microcapillary electrophoresis chip. This methodology is applicable to any device that requires multilayered PDMS, multiple alignment processes, selective PDMS bonding, and multilayered integration with downstream sensing systems. Along with the detailed step-by-step process, we discuss essential quality assurance checks that can be performed throughout fabrication to assist in troubleshooting and maximizing chip yield.

• Comprehensive instructions for designing and fabricating a programmable microvalve array.
• Selective bonding of PDMS and glass by microcontact printing.
• Numerous quality control procedures to boost chip yield.

## Graphical abstract

**Figure:**
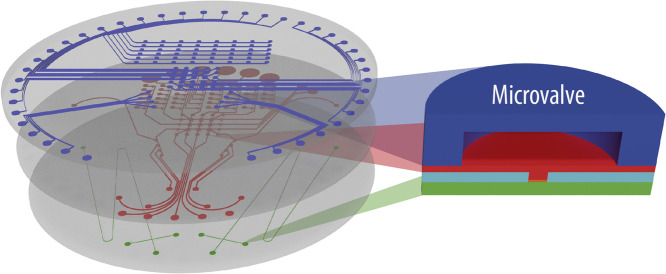

Specifications table

**Table:** 

Subject Area:	Chemical Engineering
More specific subject area:	*Microfluidics*
Method name:	Engineering design and Microfabrication of a programmable microvalve array
Name and reference of original method:	Kim, J., et al., *Universal microfluidic automaton for autonomous sample processing: application to the Mars Organic Analyzer.* Anal. Chem., 2013. **85**(16): p. 7682-8 [1]. Jang, L.W., et al., *A fully automated microfluidic micellar electrokinetic chromatography analyzer for organic compound detection*. Lab Chip, 2016. **16**(18): p. 3558-64 [2]. Shebindu, A., et al., *A fully integrated isotachophoresis with a programmable microfluidic platform*. Talanta, 2021. 225: p. 122039 [3].
Resource availability:	*SU-8 2035 (Kayaku Advanced Materials)* *Polydimethylsiloxane (Ellsworth Adhesives)* *Trichloro(1H,1H,2H,2H-perfluorooctyl) silane (Sigma-Aldrich)* *Sulfuric Acid (Sigma-Aldrich)* *Hydrogen Peroxide (Sigma-Aldrich)* *UV Exposure System (Kloe)* *Oxygen Plasma Chamber (Plasma Etch)* *Parylene coater (SCS system, KISCO Inc.)* *Motorized XYZθ Stage (Optimal Engineering Systems, Inc.)*

## Method details

### Background information

A programmable microvalve array (PMA) manipulates fluids for critical sample processing operations such as metering, transporting, or combining chemicals or analytes [1], [2], [3], [4], [5], [6]. The PMAs can run a wide variety of experiments by simply configuring the actuation sequences without altering the hardware. In the past, PMAs were utilized in wide range of applications, such as organic molecule detection [1,^2^,7], sample preconcentration [3], and organ-on-a-chip devices [8]. Each of these devices, however, needed fabrication procedures that limited the size, density, or utility of the microvalves. Furthermore, an ideal microfluidic device would be simple to integrate with downstream analyzers, including all operational procedures from samples to detection. For example, an amino acid sample is loaded into the inlet of a PMA, and it is automatically mixed with several buffers and fluorophores to identify the amino acid sample's composition and chirality. However, there are several design, protocol, and technical issues with pumping performance, material handling and treatment, and integration.

Understanding the mechanism of microvalves and the fluidic resistance of the microvalve network is crucial for PMA design. The microvalves can be modeled as thin elastic membranes, and membrane theory can be utilized to predict the performance of the microvalve. Furthermore, the fluidic resistance of the PMA network affects the dynamic operation of the PMA, hence these theoretical elements must be considered. Once a PMA is designed, it is manufactured via soft lithography. Two master SU-8 molds are used to make PDMS replicas of fluidic and pneumatic layers [9]. The fluidic layer, which consists of a microvalve array and interconnected fluidic channels, is 200∼300 um thick. Therefore, the thin PDMS film is often spun on a silane or parylene-coated SU-8 mold to avoid damage. The pneumatic layer, which is connected to the fluidic layer, regulates the actuation of the microvalve array. Normally closed valves are often chosen to ensure a strong seal. However, this type of microvalve needs a selective bonding procedure to keep the valve seat structure from sticking to the substrate [5,[10], [11], [12]. Thus, in order to produce the desired PMA, these design and microfabrication characteristics must be carefully evaluated to ensure high consistency in PMA manufacturing.

Additionally, PMAs are frequently integrated with a downstream detection system to create a completely automated platform for biological and chemical sensing. A microfluidic capillary electrophoresis (µCE) is a widely used sensing technique for the highly sensitive detection of amino acids or other organic compounds [2,[13], [14], [15]. However, the µCE requires extensive sample preparation in order to label organic molecules and mix in reagents. By combining µCE and a PMA, we are able to construct a completely automated microfluidic organic analyzer capable of doing all necessary sample manipulation, such as metering, transporting, mixing, and dispensing samples and reagents. A major consideration during the integration of PMA (PDMS) with µCE (glass) is alignment due to shrinkage. PDMS shrinks after curing, so a port connecting to an integrated µCE chip must be designed precisely considering the shrinkage ratio of the PMA [16]. Typically, the shrinkage ratio depends on the ratio of PDMS and the curing temperature, so these parameters should be precisely considered to obtain the necessary tolerances for successful integration. Thus, to have a reliable PMA and an integrated PMA platform, detailed design parameters and fabrication procedures should be introduced and discussed.

This method article outlines the complete fabrication process, highlighting the steps important to successful and consistent integration. A detailed discussion of design considerations, mold fabrication, a consistent selective bonding procedure, and alignment considerations for successful integration with a variety of tools are covered in detail. Furthermore, to enhance PMA production capability, examples of quality assurance steps and a plan for addressing potential issues will be discuss along with functional demonstration of PMA. The PMA fabricated by this method can be utilized in a wide range of fields from space exploration [17] to low-volume blood testing [18].

## Design considerations

A number of parameters must be considered when building a Programmable Microvalve Array (PMA). The size, number, and arrangement of microvalves are the key parameters to determine the performance of PMA. First, the microvalve size should be determined based on the desired volume and fabrication restrictions. Since the microvalve has a thin elastic membrane, membrane theory can be used to understand the deflection profile by considering the height of the pneumatic chamber. The theoretical deflection of the membrane can be calculated using an analytical model with the membrane theory described in Eqs. (1)–(3). Eq. (1) can be solved for maximum deflection ($\lambda_{0}$) and plugged into Eq. (2) to get the touching radius of the membrane against the top of the pneumatic chamber ($a_{b}$). Finally, the membrane deflection (λ) can be found with Eq. (3) and integrated to obtain the theoretical displaced volume of the microvalve [11,^19^,20]. All equations can be calculated with these design parameters and material properties: applied pressure (Δp), membrane radius ($a_{0})$, residual stress ($\sigma_{0})$, membrane thickness (*t*), the pneumatic chamber height ($h_{p})$, Elastic Modulus (*E*), and Poisson's ratio (υ).

(1) $$0=\frac{4t\sigma_{0}}{a_{0}^{2}\Delta p}\lambda_{0}+\frac{8Et}{3a_{0}^{4}\Delta p\left(1.026-0.793\upsilon -0.233\upsilon^{2}\right)}\lambda_{0}^{3}-1$$

(2) $$a_{b}=a_{0}\sqrt{1-\sqrt{\frac{h_{p}}{\lambda_{0}}}}$$

(3) $$\lambda \left(r\right)=\left\{ \begin{array}{c} h_{p},\;\;\;\;\;\;0<r<a_{b}\\ h_{p}\left(1-{\left(\frac{r-a_{b}}{a_{0}-a_{b}}\right)}^{2}\right){}^{2},\;a_{b}<r<a_{0} \end{array}\right.$$

**Fig. 1:**
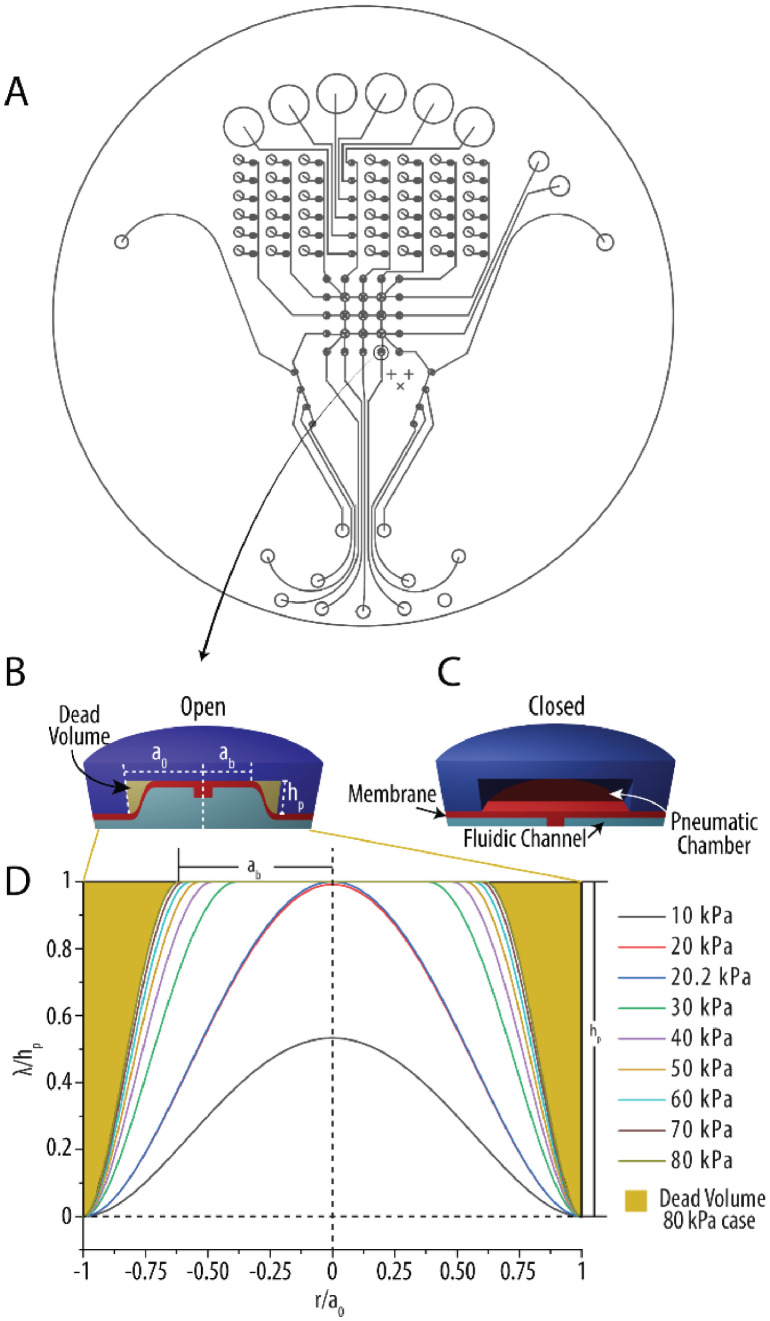
Different microvalve settings based on the pneumatic state of the device. (A) An example design of the fluidic layer of a PMA. (B) The state of a microvalve when open or under vacuum. (C) The state of the microvalve when closed or under pressure. (D) Plot of the deflection of a PDMS membrane under various vacuum applications. The pneumatic height (g) for all cases and the touching radius ($a_{b}$) and dead volume for the 80 kPa vacuum are also shown. The pneumatic height was 80 µm for this plot as well as a membrane thickness of 160 µm, a valve radius ($a_{0}$) of 0.75 mm.

Fig. 1 illustrates the different states of the microvalves depending on the pneumatic state of the PMA. The microvalve is normally closed under neutral position as described in Fig. 1B. By applying the vacuum, the membrane containing gate structure lifts up to open the microvalve. With a radius of 0.75 mm, a thickness of 160 um, and a pneumatic height of 80 μm under various applied vacuums, the theoretical deflection of the membrane was calculated as described in Fig. 1D. The peak deflection of the membrane increases as vacuum level rises. Once the membrane touches the top of the pneumatic chamber (∼20.2 kPa), the touching radius of the membrane expands as the vacuum level increases. As depicted in Fig. 1D for the 80 kPa vacuum case, there is small amount of dead volume at the corner of the pneumatic chamber due to geometrical constraint. By considering the dead volume, a theoretical displaced volume for each valve opening action can be derived by integrating Eq. (3) and confirmed with actual volume empirically.

Once the size of the microvalve is determined using all design criteria, the array of microvalves is designed to generate the PMA. In practice, the overall performance of a PMA will be influenced by the fluidic resistance of any pumping path defined by Eq. (4), and the actuation time interval between microvalve actuations will need to be tuned for maximum performance as defined by integrating Eq. (3).

(4) $$R=\frac{12\mu L}{wh_{f}^{3}F}$$

L is the length of the fluidic path, w denotes the channel width, $h_{f}$ denotes the channel height, F denotes the force acting on the fluid, and µ is the dynamic viscosity. This fluidic resistance between microvalves has an impact on the time required for actuation (the time between each microvalve operation). Adequate actuation time that enables complete emptying or filling of a microvalve should be precisely determined in order to achieve high pumping efficiency. Otherwise, not only will the valve not work as expected according to Eq. (3), but the PMA often develops bubbles during pumping sequences.

### Fabrication, packaging, and surface modification of PMA

#### Fabrication of pneumatic and fluidic molds

The fluidic and pneumatic layers are designed in AutoCAD (Autodesk Inc.), converted to photomasks, and fabricated using a standard soft-lithography technique. Briefly, two five-inch silicon wafers (one for the pneumatic mold and one for the fluidic mold) are dehydrated on a hotplate set to 95°C, placed in a spin coater, then SU-8 2050 is poured on top and spun to the desired thickness (∼80 µm for pneumatic, ∼50 µm for fluidic). The wafers are then removed from the spin coater and placed on a hotplate for the pre-exposure bake (10 min at 65 °C then 16 min at 95 °C). UV exposure is completed using the designed photomasks with a dose of 240 mJ/cm^2^ (UV KUB2), followed by a post-exposure bake (5 min at 65 °C then 10 min at 95 °C) before developing the wafer for 4 min and completing a hard bake at 150 °C for 30 min. Prior to use of both molds, the geometry of each fluidic and pneumatic layer is measured with a surface profiler and both molds are inspected visually under a microscope.

#### Parylene coating on a fluidic mold

Since the fluidic layer of the PMA is thin (usually between 100 µm and 300 µm of PDMS), there is a risk of damage to the layer when removing it from the fluidic SU-8 mold. To address this, the fluidic mold is coated with parylene-C to achieve a thickness of less than 1 µm utilizing a Specialty Coating Systems PDS 2010 and a parylene adhesion promoter (Silane A-174) after visual inspection under a microscope. The parylene acts as a surfactant, reducing the amount of pulling force required for detaching the thin PDMS and, contrary to conventional silane coatings, provides visual cues to any damage or wear the coating may have.

#### Preparation of PDMS replica

**Fig. 2:**
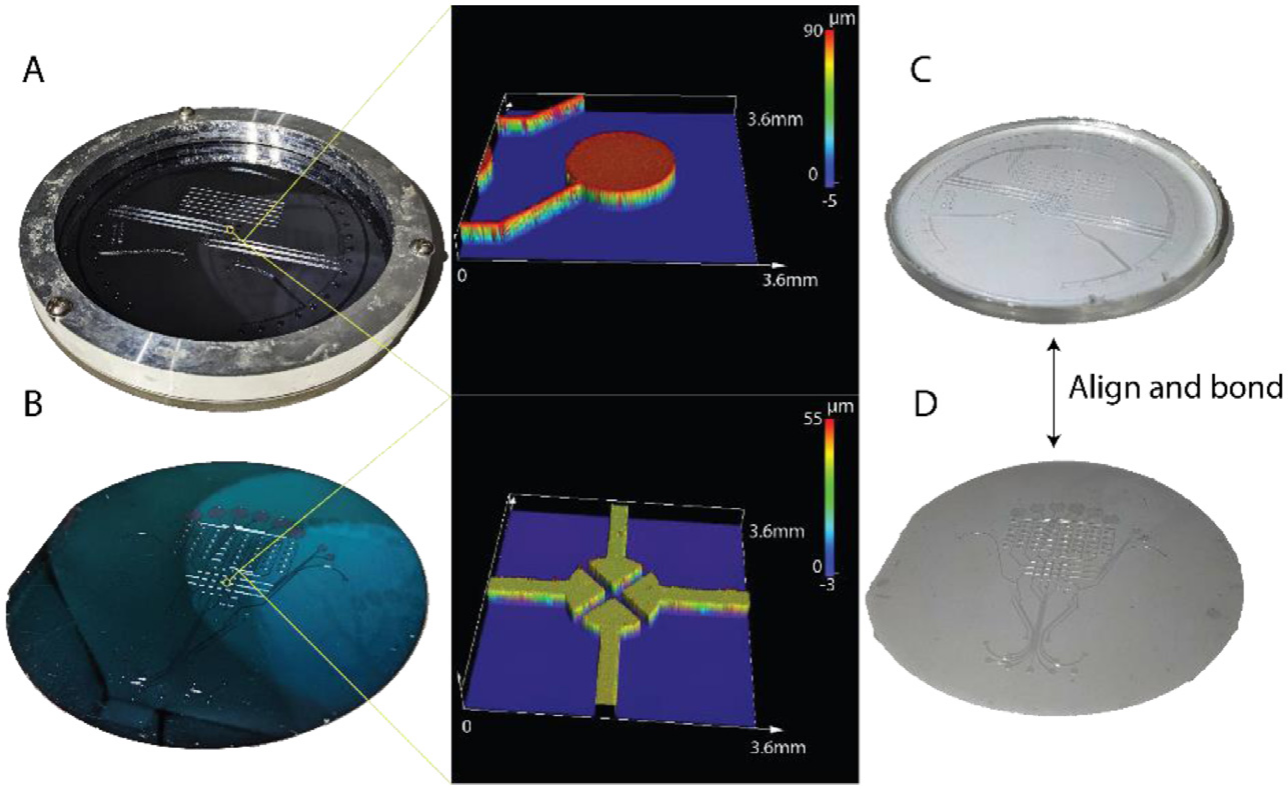
The two molds for a PMA design with optical profilometer images of the mold quality. (A) Pneumatic mold in a metal bath. The bath ensures that a consistent thickness of PDMS can be obtained while also allowing for easy removal. The profiler image shows the uniformity of the pneumatic channel at 80 µm and the image on the right is of the PDMS replica. (B) The parylene coated fluidic mold. The coating acts as a surfactant, making removal of the thin PDMS films simple without decreasing the quality of the mold itself. The surface profiler shows that the addition of parylene does not reduce the resolution or aspect ratio of the mold. (C) The PDMS replica of the pneumatic layer after removal from the mold is bonded with (D) the PDMS replica of the fluidic mold after plasma exposure and alignment.

Pouring begins with the preparation of PDMS (10:1 ratio) and its degassing in a vacuum chamber. The PDMS is then put into the pneumatic bath (Fig. 2A) and degassed again. The remaining PDMS is put onto the fluidic wafer (Fig. 2B) during the degassing stage for the pneumatic layer and spin-coated at 200 RPM for 30 s to achieve a 200 µm thick PDMS. Both the fluidic and degassed pneumatic layers are then placed overnight on 45°C hot plates to minimize PDMS shrinkage [16]. PDMS typically shrinks 2∼10% depending on curing temperature. Overnight curing at 45°C results in ∼0.3 % shrinkage which is within tolerable limits for integration.

#### Bonding of pneumatic-fluidic PDMS layers

The procedure of bonding two layers begins with the removal of the PDMS from the two molds. To prepare the pneumatic PDMS layer a sharp knife was used to cut around the circumference of the molding bath. The layer is removed from the mold using the knife as a lever to peel the PDMS off the mold, and the layer is wrapped in plastic wrap and stored until use (Fig. 2C). To prepare the fluidic PDMS layer, two pieces of polyethylene terephthalate (PET) plastic sheet slightly larger than the fluidic mold are prepared to aid in removing the thin PDMS from them mold. The first piece of plastic is placed on top of the PDMS layer on the fluidic mold, and the PDMS is removed from the fluidic mold and transferred to the plastic film using flat tip tweezers. The tweezers can scratch the parylene coating on the mold over time, however the adhesion promotion step performed during the parylene deposit will prevent the parylene from flaking off. Once the PDMS has been entirely removed from the mold, residual stress from the PDMS stretching can remain. This is mitigated by picking up one side of the PDMS, slowly peeling it away from the plastic film until approximately halfway across, and then slowly lowering it back to the plastic. The second plastic film is now placed on the exposed side of the PDMS until bonding preparation begins (Fig. 2D).

**Fig. 3:**
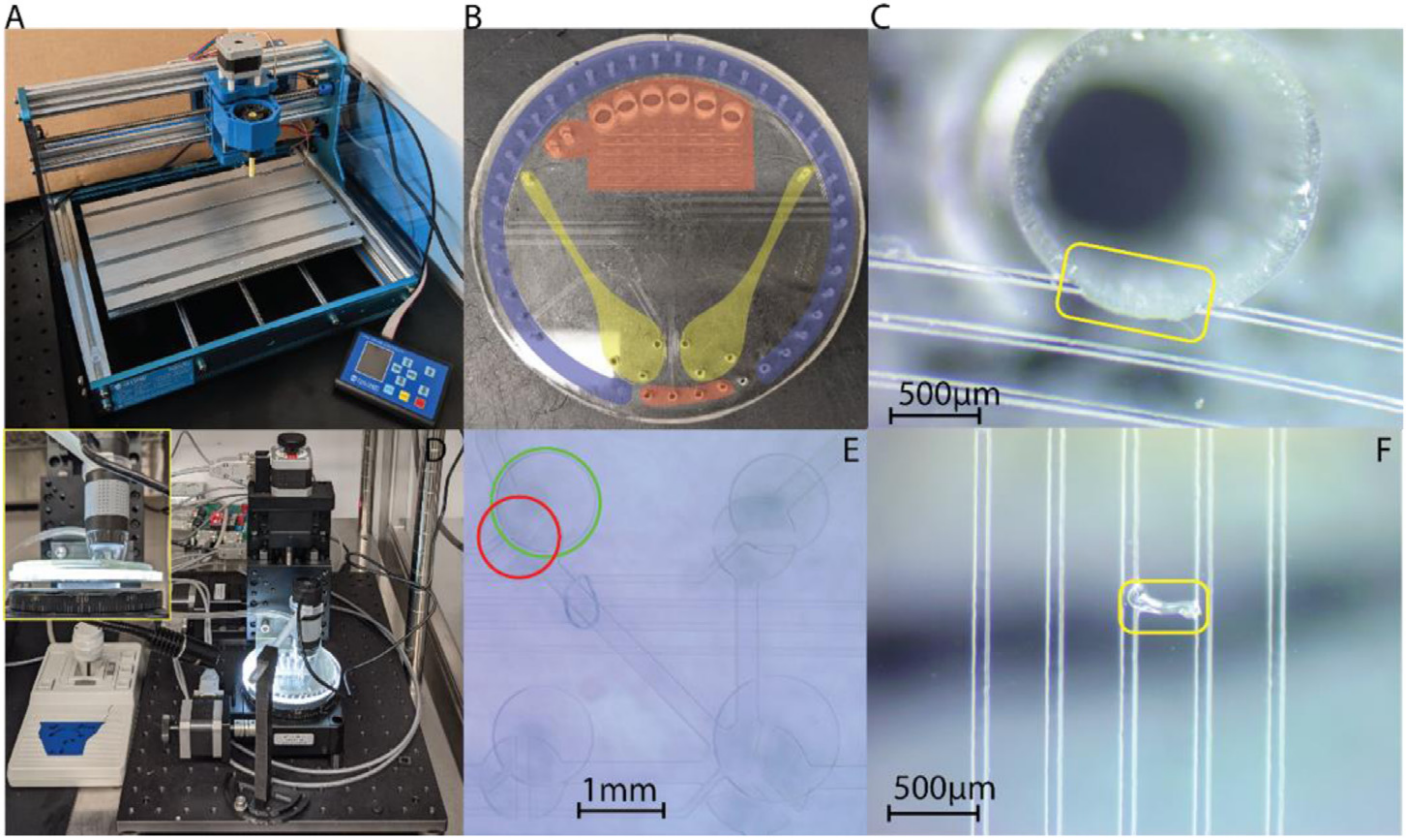
Images of the different motorized systems used during punching and alignment as well as the regions that need to be punched and potential failures that can occur during punching and alignment. (A) CNC punching system used to accurately and consistently punch out all interfacing ports and wells. (B) Overview of a PMA design showing locations of pneumatic ports (blue), fluidic ports (red), and CE interface ports (yellow). Because the pneumatic ports will interface with a manifold and the CE ports will interface with a µCE chip, they are punched into the pneumatic layer prior to bonding to the fluidic layer with the CNC punching system. (C) Misaligned punching can result in pneumatic line failure due to the punch severing the pneumatic line. (D) Motorized xyzθ alignment system used for all alignment steps. The inset image is a detailed front view showing the vacuum chuck. (E) An example of what is considered during alignment. The red circle should be completely inside the green circle, and with perfect alignment, they would be concentric. This is checked at multiple locations around the chip before bringing the two PDMS pieces into contact. (F) Contamination bridging two pneumatic lines after bonding the pneumatic and fluidic PDMS layers.

Once both PDMS replicas are removed from the molds, the pneumatic and fluidic access ports are punched into the pneumatic layer (Fig. 2C) prior to bonding. A computer numerical control (CNC) punching system, as shown in Fig. 3A, is used to avoid any misalignment issues caused by shrinkage and to ensure the orthogonality of the ports. Poor orthogonality can often cause interfacing issues with both actuation manifolds and essential integration features. To start the punching process, double-sided tape is put on a PDMS holder with a pre-defined reference position. Using the locations of multiple alignment marks and the valves, the holder is brought into contact with the pneumatic layer. The holder is then secured to the bed of the CNC puncher and the reference position is registered into the punching software before starting the software and allowing all of the interface features to be punched. After punching the 48 pneumatic and interface holes shown in Fig. 3B, the pneumatic layer is immediately cut down to size using a circle cutter. After cutting, the thickness is measured in four locations around the layer to ensure uniformity, and the layer is then wrapped in plastic wrap until bonding starts. As shown in Fig. 3C, pneumatic line breakage can occur as a result of manual punching or improper alignment of the pneumatic layer in the CNC.

For final preparation for the pneumatic and fluidic bonding, the fluidic layer needs to be smoothed, both layers must be cleaned, and practice alignment should be done. First, the plastic film piece is removed from the unfeatured side of the fluidic PDMS. To remove any trapped air, the PDMS layer can be lifted from the edge to the area around the bubble and then slowly placed back down. Several bubbles may exist as a result of particles trapped beneath the PDMS, which must be cleaned using tape before proceeding with the bonding procedure. If bubbles are not effectively eliminated, the fluidic layer may deform unexpectedly during plasma exposure. Finally, particles are removed from the top of the fluidic layer using tape. Dust causes poor bonding and valve failure, thus cleanliness during the bonding stage is crucial. After that, the fluidic layer is covered with a petri dish and placed away during pneumatic cleaning. For the pneumatic PDMS, the cleaning process starts with a rinse in isopropyl alcohol (IPA) and deionized (DI) water to eliminate any remaining materials, followed by drying with N_2_ gas, dehydrating on a hot plate set to 45°C, and a final cleaning with tape.

Prior to irreversible bonding, alignment practice should be done with the layers to facilitate quick and accurate alignment once plasma exposure is completed. First, the pneumatic PDMS is removed from the hotplate and allowed to cool to room temperature. A vacuum chuck is used to secure the pneumatic PDMS while the fluidic layer is placed on the base of a motorized alignment stage. Next, a digital microscope (Plugable Technologies, USA) is used to align multiple microvalves as shown in Fig. 3D. Because the PMA is 100 mm in diameter, small local translational or rotational adjustments will lead to improper alignment in all areas of the chip, as shown in Fig. 3E. To verify appropriate alignment, at least two valves and two alignment markers on each end of the PDMS layers should be inspected. If regions of the fluidic PDMS are misaligned despite being perfectly aligned in other areas, the fluidic PDMS is likely still stretched. Thus, the stretching relaxation approach stated previously should be repeated before practice alignment proceeds. After checking the alignment marks and microvalves, the pneumatic PDMS is pressed into the fluidic PDMS layer. This should form a weak, reversible, bond between the two layers without outside force application. If bubbles form, the region is inspected for any contaminants following the separation of the assembled piece. This procedure should be repeated until the alignment practice is completed quickly and each layer is guaranteed to be clean. Irreversible bonding of the pneumatic and fluidic layers then proceeds as normal with the plasma set to 80% power and an O_2_ flowrate of 7.5 sccm. Both PDMS layers (Fig. 2C and D) are placed into the chamber with the fluidic layer set PDMS side up (channel side down) and the pneumatic layer channel side up. Both layers were exposed simultaneously for 30 s, removed from the chamber, sprayed with N_2_ briefly to remove dust, and alignment is done as outlined above. After bringing the layers into contact and removing any air bubbles, the layers are placed on a hot plate at 65°C for 30 min.

Once the fluidic and pneumatic layers are assembled, the fluidic wells and interfacing holes are punched indicated in Fig. 3B. To punch all wells and holes with proper alignment, the pneumatic holes are used as guides. Positions of critical interfaces, like the wells connecting to a µCE chip shown in Fig. 3B, should be double checked before proceeding. In the last step of cleaning, the PMA is rinsed with IPA and DI water, then dried with N_2_ gas and put on a hot plate set to 45 °C for about 10 min. The PMA is wrapped in plastic wrap and stored until needed.

#### Verification of PMA

Once bonding has finished, the extra PDMS at the perimeter of the fluidic layer is trimmed and the combined pneumatic-fluidic PMA is removed from the lower plastic covering. Next, the function of all microvalves is verified by connecting vacuum and pressure sources to all the pneumatic ports. If the microvalves do not stay actuated under vacuum, there is likely contamination branching two or more pneumatic lines. If a valve does not actuate at all, either the pneumatic mold is damaged, or contamination has blocked the line. Any failure at this stage prompts an investigation of both the PMA and pneumatic mold under a microscope. This identifies if the issue is one-off, i.e. dust contamination, or a more serious issue, i.e. a line broken on the mold. Fig. 3F shows an example of dust bridging two pneumatic lines. This would be due to poor air quality in the fabrication environment or insufficient cleaning of the layers before the bonding of two PDMS layers. A break in the pneumatic mold caused by an error during the SU-8 process or damage from rough storage is also possible. This would be seen as a microvalve that does not actuate across multiple devices if not investigated after an initial failure. Fig. 3C shows a port being punched through a pneumatic line. The likelihood of this happening depends greatly on the chip design, but the alignment of all punching guides should be carefully checked to look for pneumatic lines in the punch path prior to actual punching.

#### Surface passivation

After completing the PMA, the PMA can be bonded to either blank glass or a CE glass chip. However, if the entire surface of the PMA is activated with oxygen plasma, the gates of microvalves will bond to the surface, resulting in sticking issues. A selective bonding strategy is necessary to avoid unwanted bonding of the PMA gate structures to the underlying the glass substrates. In our approach of selective bonding, we utilize a unique microcontact printing technique to passivate the gate [21]. This approach utilizes Perfluorooctyl-Trichlorosilane (PFTCS, CAS: 78560-45-9) that has been placed on a masked PDMS stamp and pressed onto the gate structures prior to bonding. The transferred PFTCS acts as a passivator for the stamped region, preventing gates from bonding to the glass substrates. First, a patterned tape mask, containing circles representing the microvalve array is created using an AutoCAD. To prevent PFTCS stamping outside of the microvalve area and causing leakage, the circles have a 0.1mm smaller diameter than the size of the microvalve. Additionally, to accommodate for PDMS shrinkage, the design is scaled to 99.7% of its original size around the center of the PMA. The mask is then cut with a vinyl tape cutter (Roland, USA) and placed on a 3 mm thick PDMS slab [22]. The stamp is then taped to the top of a vacuum desiccator (Bel-Art, USA). A heat block is heated to 115°C for at least 2 h, and the lids of two microcentrifuge tubes are cut off to retain the PFTCS solution. The heat block is then placed at the bottom of the desiccator, followed by the lids with 100 µL PFTCS apiece. The desiccator is then secured and pumped down to -80kPa before being sealed. To achieve appropriate PFTCS deposition, the chamber is left under vacuum for 30 min.

**Fig. 4:**
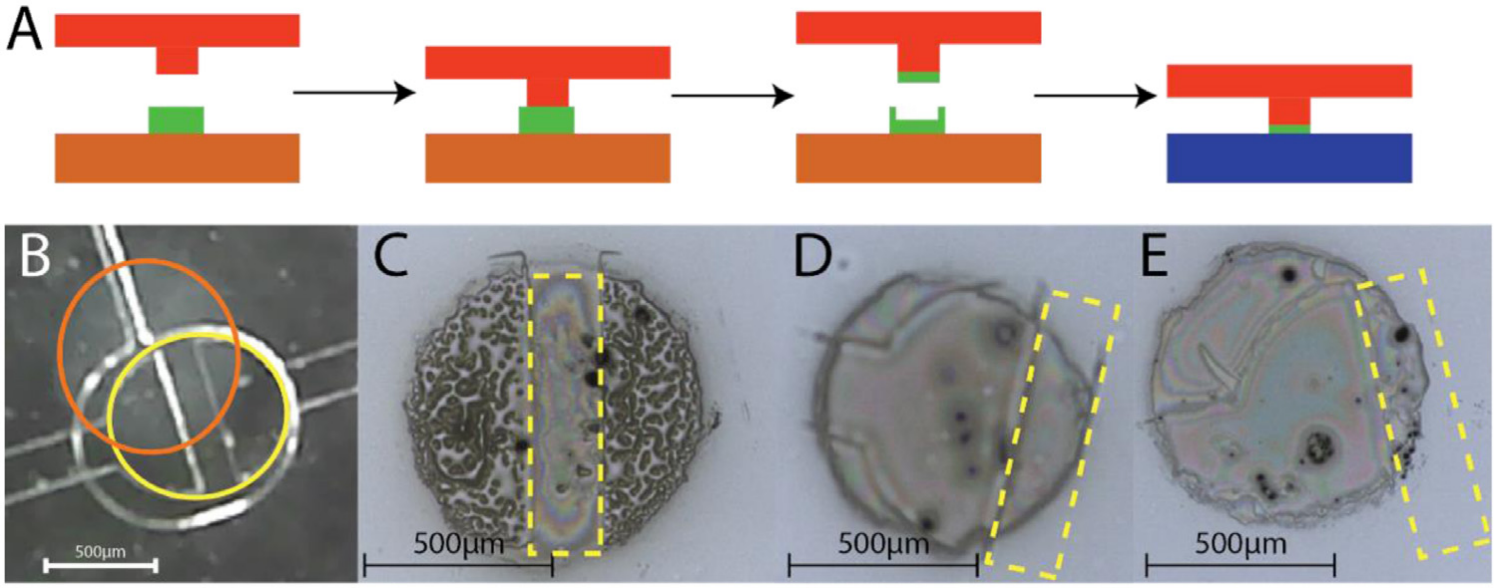
Explanation and results from PFTCS stamping. (A) A brief overview of how the stamping process works. First, the valve (red) is brought into contact with PFTCS (green) on a stamp (orange). When the valve is removed, it retains some of the PFTCS and this passivates its surface during bonding to glass (blue). (B) A view of the PMA and stamp during alignment. The orange circle (the PFTCS) should be concentric with the yellow circle (the microvalve). (C–E) Different quality of PFTCS transfer viewed under 5x magnification with the yellow outline denoting the PDMS gate. (C) Desired Transfer, (D) Partially Stuck Valve, (E) Valve Failure.

After the appropriate deposition period has passed, the stamps are removed from the vacuum chamber and placed in a petri dish covered to prevent contamination. To precisely stamp PFTCS onto the gate of microvalves, the motorized alignment stage is used again. First, the cleaned PMA is placed into the vacuum chuck with the channel side facing down from the chuck. The chuck is then secured to the motorized stage and the stamp is placed on the rotating stage PFTCS up after removing the tape mask. Using the moveable digital microscope and the motorized controls, the PFTCS spots are aligned with the gates and pressed together. Any rotational or translational misalignment can lead to valve sticking so, like bonding, multiple valves are checked before and after pressing the PMA and stamp together to verify alignment. The PMA with the stamp is then placed on a hotplate set to 150 °C with a 3 kg weight to promote the condensation reaction and conformal contact. After 30 min, the weight and stamp are removed from the PMA and placed in the petri dish for storage. The PFTCS stamps can be utilized for confirmation of PFTCS transfer using a high-resolution microscope or optical profilometer. Fig. 4A illustrates a cross section of the PFTCS and gate prior to and following stamping. Each gate should leave a noticeable mark on the PFTCS, as illustrated in Fig. 4B–D; if this is not the case, final bonding will almost certainly result in the valve not operating correctly. The desired stamp pattern, shown in Fig. 4B, is a clear imprint of a gate in the center of the PFTCS design. Fig. 4C and 4D highlight the consequences of mismatched gates. Because at least a portion of the gate is stamped all the way across in Fig. 4C, that microvalve is expected to function normally but with reduced performance due to the valve not fully opening. In comparison, Fig. 4D depicts a microvalve that lacks a segment all the way across, indicating that it will likely not operate in the final product. Finally, the PMA is allowed to cool to the ambient temperature gradually prior to final bonding.

#### Final bonding

The final bonding takes place in a similar way to that of conventional glass-PDMS bonding. To prepare the bonding PMA to a featureless glass substrate, a piranha acid (2 H_2_SO_4_:1 H_2_O_2_) bath and a DI water bath are prepared with the glass substrate being submerged in the piranha acid. After five minutes, the glass substrate is removed, briefly washed with DI water, and then placed in the water bath for ∼30 s. The glass is then removed from the DI water and N_2_ gas is used to dry the substrate. After that, the glass substrate is dehydrated at 95°C for 10 min and then allow to cool to room temperature before bonding.

**Fig. 5:**
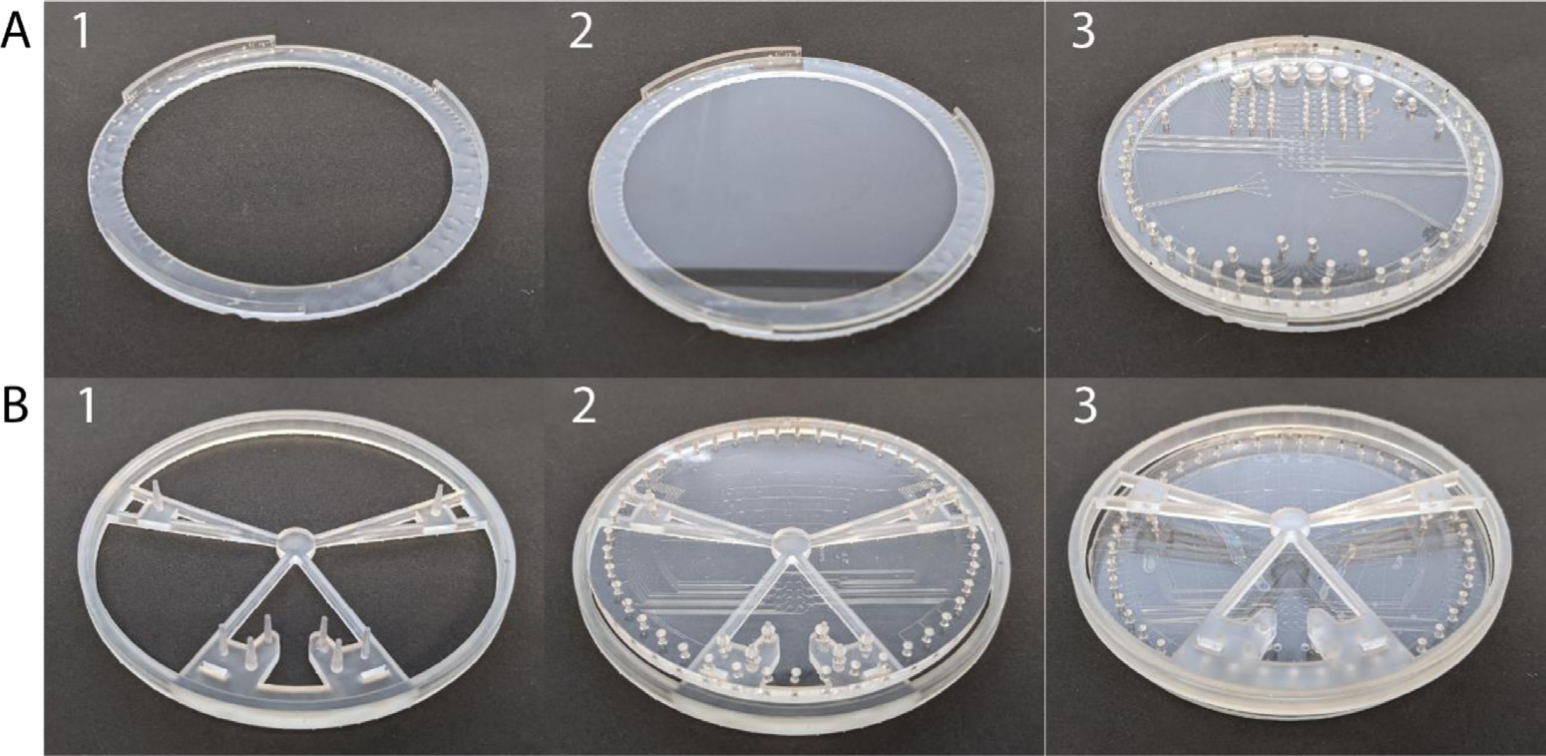
Bonding guides and steps for (A) bare glass and (B) featured glass. Bare glass is placed into the wafer holder and the lips on the wafer holder are used to center the PMA on the glass wafer. The interface holder in (B) is fed through the interface holes on the PDMS side and then the posts are used to locate those holes in the featured glass.

After the PMA and a glass substrate have cooled down, the glass substrate is placed into a wafer holder with a lip to accept the PMA (Fig. 5A1). As previously noted, tape can be used to clean the perimeter of the PMA, but it should not be used near microvalves unless absolutely essential to remove stubborn dust. Next, the glass and PMA, with fluidic side up, are placed in the oxygen plasma chamber for surface activation. Both PMA and the glass substrate are then removed from the chamber after oxygen plasma treatment and briefly cleaned with N_2_ gas. The PMA is positioned channel side facing the glass using the holder (Fig. 5A3) to ensure that the PMA is centered on the glass before being placed onto the glass. Pressing the two together, the PMA and glass are carefully removed from the wafer holder and any remaining air bubbles are pushed towards the perimeter of the chip. Finally, the chip is placed onto a hotplate at 65°C for 30 min to promote bond strength. After cooling the chip, it is bonded and ready for final quality assurance testing.

For bonding to a featured glass like a µCE chip, slight modifications to this protocol are required to ensure perfect alignment at the interface points. In this case, the PMA is held by an interface holder (Fig. 5B1) with posts that go through the interface points of the PMA and fit into the wells in the featured glass chip. The featured glass is placed into the plasma chamber with the PMA fitted into the interface holder with channels facing up. After plasma exposure, both are removed from the chamber and the PMA is aligned to the featured glass chip using the posts. The PMA is pushed off of the posts and placed onto the featured glass and the interface holder is removed. Any remaining bubbles are pushed to the outside of the chip and the PMA and featured glass are placed on a hotplate at 65 °C for 30 min to promote bond strength.

It should be noted that approximately one minute after removal from the plasma chamber, the bond strength of any exposed areas is drastically reduced and most likely will not bond. As a result, the bonding procedures should be completed immediately to ensure adequate bonding. Furthermore, since both the glass and PMA are easily detachable after initial contact, the tolerance of the interface holder should be carefully evaluated such that both PMA is easily removable. For a four-inch diameter PMA, one percent tolerance allows for a satisfactory fit to hold, bond, and release the PMA after bonding procedures.

### Final quality check and validation of PMA

#### Testing bed hardware

**Fig. 6:**
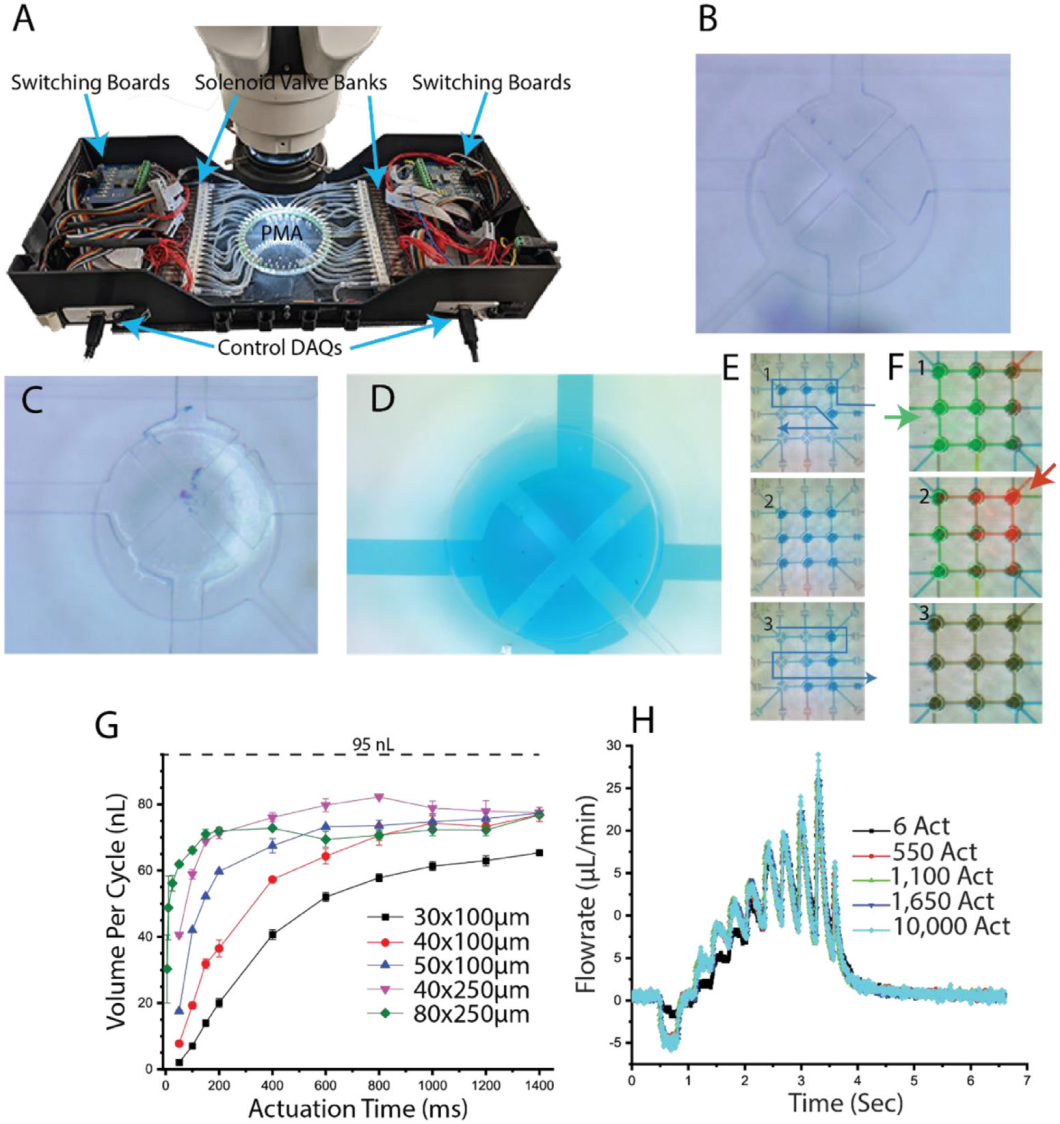
Final QA setup and functional results of chip testing. (A) A testing bed for final quality assurance testing and performance evaluation. The switching boards control the solenoid valves based on the digital output of the NI-DAQs at the bottom of the figure. The changes in the solenoid valves correlate with state changes on the PMA. B and C) Examples of stuck and functional valves, respectively. (D) An example of the delamination of the PDMS from the glass. The dyed water is visible outside of the valve and fluidic lines, meaning that pressure has forced fluid to separate the PDMS and glass. (E) An example of a pumping sequence in which the nine central valves are opened one at a time while one external valve is open to pull in fluid (1). Once all of the central valves are full (2) a different external valve is opened, and the original is closed. The central valves then close pushing fluid out the open external valve (3). (F) Example of mixing of two fluids in the PMA. A sequence similar to that described in B is used to alternatively pull in green (1) and red fluid (2). The resulting mixture is shown in 3. (G) Measurement of the volume per cycle per valve of the pumping sequence described in B with different fluidic resistances and actuation times, which is the time between valve operations in the sequence. The lower fluidic resistance chips (40 µm x250 µm and 80 µm x250 µm) reached the saturation point for their volume per cycle at a much lower actuation time than the other cases. This allows for quicker cycles on the chip while maintaining bubble stability. The dotted line at the top corresponds to the predicted performance of these valves (0.75mm radius, 160 µm thick membrane, 80 kPa vacuum applied), showing a 15% difference between the max real-world performance and the predicted value. (H) Plot of the pumping rate for a completed PMA, running the sequence described in B, comparing the performance of a chip over 20 h of continuous use. The chip did not generate any bubbles and ran without human intervention for the entire time period. The actuations listed are for a single valve in the 11-valve sequence.

A testing bed for the PMA platform can be fabricated with digital switching boards (ULN2803 board, Elexeol), solenoids (S070M-6BG-32, SMC), a controller (typically a NI-DAQ or an Arduino), and vacuum and pressure lines along with a manifold or barbed fittings for interfacing with the chip. Fig. 6A shows an example of a testing bed. The given components and layout are suggestions, and any testing bed that allows for changing the pneumatic states of each of the pneumatic inlets will allow for functional testing.

#### Assembled PMA quality assurance (QA)

General QA is completed throughout the fabrication process. Final QA is performed by setting up the PMA in a testing bed shown in Fig. 6A. First, all microvalves are opened and the chip can be visually inspected for sticking microvalves. The operation of the PMA can then be tested by establishing a sequence of microvalve actuations to transport fluid from any inlets to any outlets. If microvalves become stuck, as shown in Fig. 6B, they may need to be placed under vacuum for an extended period of time, but more than likely, a new chip will need to be fabricated with careful attention given to the surface passivation. The open valve in Fig. 6C is distinguished from the stuck valve by the apparent distortion of the microvalve membrane within the pneumatic channel. Fig. 6D also depicts a microvalve with evidence of delamination of the PMA and glass. The presence of colored liquid outside of the microvalve and fluidic lines indicates that pressure applied from another valve or external source has separated the PDMS and glass. This can be caused by a variety of factors, the most likely of which is contamination of the glass and/or PMA prior to bonding. This weakens the PDMS-glass bond, increasing the probability of failure.

#### Validation of pumping and programming performance

The testing bed is used with valve actuation sequences to move fluid around the chip, demonstrating both the pumping capability and usability in real-world experiments. To evaluate pump performance, a PMA equipped with a 3 × 3 microvalve array and perimeter Input/output microvalves was successively fabricated to transport liquid from an inlet to an outlet indicated in Fig. 6E. In brief, an inlet valve is opened first, followed by the sequential opening of nine pumping valves. After that, an outlet valve is opened and each of the pumping valves is closed, allowing the fluid to flow. The outlet is linked to a flow meter (SLI-1000, Sensirion), and the instantaneous volumetric flow rate is determined across three pumping cycles. After integrating the flow rate data for each cycle, the volume per cycle is normalized based on the number of pumping valves. In an ideal world, this volume every cycle would equal the theoretical volume obtained by integrating Eq. (3) with the microvalve geometry (0.75 mm diameter, 80 µm pneumatic height, and 160 µm thickness), which is around 95 nL. However, due to a number of factors not considered in the theoretical calculation, as well as the channel's fluidic resistance, the real volume of each cycle will vary. The results of the pumping performance validation and the impact of Eqs. (3) and (4) on chip performance are shown in Fig. 6G. The legend indicates different channel heights (30, 40, 53, and 80 µm) and heights (100 and 250 µm), as well as the volume of each cycle per microvalve for each case. The examples with the lowest fluidic resistance (40 µmx250 µm and 80 µmx250 µm) both achieve the maximum volume per cycle at 200 ms actuations, but the other cases require at least double the time. Additionally, Fig. 6G also shows the theoretical displaced volume for a valve as calculated by integrating Eq. (3) for the microvalve geometry. A stable volume per cycle is reached with an efficiency of 85%, and this efficiency is consistent for all four chips that reached a stable volume per cycle.

### The usability of the PMA

To demonstrate the usability of the PMA in real experiments, a mixing sequence is evaluated. Fig. 6F shows the example mixing program on the PMA. Two different colored fluids are pumped sequentially to a storage well in the sequence seen in Fig. 6F. The last color shift indicates that the two fluids have successfully mixed, and further characterization could be accomplished by diluting a fluorophore [23]. This validation is analogous to the labeling that would be necessary for real-world use of the PMA and could be modified to accommodate the required ratio or dilution without altering any hardware. Additionally, this PMA has 2^25^ distinct operational states that can be utilized in a number of sequences to perform various fluid processing or delivery functions. Finally, the robustness of the presented approach was evaluated by executing the pumping sequence from Fig. 6B for extended periods of time. The results of this validation are depicted in Fig. 6H. The flow rate capabilities of a PMA that has been continuously utilized to pump fluid for more than 20 h indicate no degradation in functionality. The PMA chip, in particular, was able to operate for this length of time without creating bubbles, which would have had a detrimental effect on performance. Additionally, this test demonstrates that there is no risk of hysteresis or performance degradation when a PMA fabricated with this method is used for an extended period of time.

## Declaration of Competing Interest

The authors declare that they have no known competing financial interests or personal relationships that could have appeared to influence the work reported in this paper.

## Data Availability

Data will be made available on request.
